# P4HA2 contributes to head and neck squamous cell carcinoma progression and EMT through PI3K/AKT signaling pathway

**DOI:** 10.1007/s12032-024-02358-w

**Published:** 2024-05-23

**Authors:** Yan-Ling Wu, Wan Liu, Tingting Zhao, Jing Jin

**Affiliations:** 1 Department of Radiation Oncology, National Cancer Center/National Clinical Research Center for Cancer/Cancer Hospital and Shenzhen Hospital, Chinese Academy of Medical Sciences and Peking Union Medical College, Shenzhen, 518116 Guangdong China; 2 Department of Head and Neck Surgery, National Cancer Center/National Clinical Research Center for Cancer/Cancer Hospital & Shenzhen Hospital, Chinese Academy of Medical Sciences and Peking Union Medical College, Shenzhen, 518116 Guangdong China

**Keywords:** P4HA2, Head and neck squamous cell carcinoma, EMT, PI3K/AKT pathway, Prognostic marker

## Abstract

Head and neck squamous cell carcinoma (HNSCC) can be defined as a deadly illness with a dismal prognosis in advanced stages. Therefore, we seek to examine P4HA2 expression and effect in HNSCC, along with the underlying mechanisms. This study utilized integrated bioinformatics analyses to evaluate the P4HA2 expression pattern, prognostic implication, and probable function in HNSCC. The study conducted various in vitro experiments, including colony formation, CCK-8, flow cytometry, wound healing, and transwell assays, on the human HNSCC cell line CAL-27 to examine the involvement of P4HA2 in HNSCC progression. Moreover, western blotting was used to investigate epithelial-mesenchymal transition (EMT) markers and PI3K/AKT pathway markers to elucidate the underlying mechanisms. P4HA2 expression was significantly enhanced in HNSCC, and its overexpression was correlated to tumor aggressiveness and a poor prognosis in patients. Based on in vitro experiments, the overexpressed P4HA2 enhanced cell proliferation, migration, invasion, as well as EMT while reducing apoptosis, whereas P4HA2 silencing exhibited the reverse effect. P4HA2 overexpression enhanced PI3K/AKT phosphorylation in HNSCC cells. Moreover, LY294002 was observed to counteract the effects of upregulated P4HA2 on proliferation, migration, invasion, and EMT in HNSCC. Collectively, we indicated that P4HA2 promoted HNSCC progression and EMT via PI3K/AKT signaling pathway.

**Supplementary Information:**

The online version contains supplementary material available at 10.1007/s12032-024-02358-w.

## Introduction

Head and neck cancers, which encompass various cancers such as nasopharyngeal cancer, oral cancer, laryngeal cancer, and hypopharyngeal cancer, can manifest with diverse primary sites and pathological subtypes. Squamous cell carcinoma is the most common type of head and neck cancer, accounting for over 90% of cases [1]. Head and neck squamous cell carcinoma (HNSCC) is considered a widely occurring malignancy on a global scale, with an excess of 800,000 incidences and over 350,000 mortalities annually [2]. HNSCC threats, to a great extent, not only human health but also their quality of life due to its elevated rates of primary-site recurrence and metastasis, as well as late-stage diagnosis and poor prognosis [3]. Although surgical technology has improved dramatically, the use of radiation and chemotherapy has demonstrated enhanced management of local HNSCC control; these treatment options can lead to cosmetic deformity and functional impairment of vital functions [4]. The low overall 5-year survival rate that is shown in HNSCC patients can be attributed to the increased treatment failure and recurrence rates, besides the nodal metastasis. Despite treatment, the five-year survival rate of patients having HNSCC persists at around 60% [5]. The onset and progression of HNSCC in humans may be determined by a variety of regulatory mechanisms, and elucidating the molecular targets underlying molecular mechanisms is critical for the prevention and treatment of HNSCC.

Numerous intricate processes are involved in the manufacture and deposition of collagen, and many post-transcription modification proteins control these activities [6]. Prolyl 4-hydroxylase subunit Alpha-2 (P4HA2), a crucial enzyme in collagen manufacture, is made up of two alpha and two beta subunits, which are identical to each other [7]. According to reports, P4HA2 causes extracellular matrix remodeling when the oxygen level is low [8]. P4HA2 was shown to accumulate in lung cancer cells that were methylated in the RASSF1A promoter and contributed to the acceleration of collagen deposition and in vivo metastatic dispersion [9]. According to Cao et al. [10], P4HA2 functions as an oncogenic agent by inducing EMT in cervical cancer cells, thereby enhancing their motility, invasion, and proliferation. Accumulating evidence has confirmed that P4HA2 overexpression in multiple cancers, including lung adenocarcinoma, prostate cancer, glioma, and cervical cancer, has a correlation to patient prognosis [7, 11]. Kisoda et al. [12]. utilized bioinformatic data mining techniques to propose that P4HA2 served as a partial EMT (p-EMT) associated gene and exhibited a significant correlation with unfavorable survival outcomes in patients with HNSCC. Nevertheless, experimental verification is still lacking regarding the role of P4HA2 in HNSCC and its association with EMT.

We aimed to analyze the expression of P4HA2 in HNSCC and assess its diagnostic and prognostic value in patients with HNSCC, as well as elucidate the potential mechanism underlying the effect of P4HA2.

## Materials and methods

### Bioinformatic analysis

The RNA-seq gene expression data and clinical records were retrieved by accessing The Cancer Genome Atlas (TCGA) dataset (https://portal.gdc.cancer.gov/), with extracting the data in the form of transcripts per million reads (TPM). The P4HA2 levels were investigated in 33 distinct human cancers, 504 HNSCC and 44 non-malignant tissues, as well as 44 HNSCC and their corresponding non-cancerous tissues, utilizing the TCGA data. The receiver operator characteristic (ROC) curve for diagnostic purposes was produced utilizing the pROC package in R. The survival package was used for performing proportional hazards hypothesis testing and fit survival regression, while the survminer and ggplot2 packages were used for visualizing the results. The correlation analysis was conducted using Spearman correlation. Genes exhibiting strong positive or negative correlation coefficients with P4HA2 were identified and chosen. Utilizing the clusterProfiler package, enrichment studies for the Gene Ontology (GO), Kyoto Encyclopedia of Genes and Genomes (KEGG), as well as Gene Set Enrichment Analysis (GSEA) were carried out. CancerSEA (http://biocc.hrbmu.edu.cn/CancerSEA/) was accessed for the detection of the probable P4HA2 role in HNSCC [13].

### Tissue samples

A total of 36 tissues from patients with HNSCC were collected from Cancer Hospital & Shenzhen Hospital, Chinese Academy of Medical Sciences and Peking Union Medical College between May 2019 and June 2021. The normal control tissue was obtained as far as possible from the tumor. The Institutional Ethics Committee granted approval for conducting this experiment, and each patient provided informed consent. After being surgically removed, the tissues were subjected to rapid liquid nitrogen freezing and subsequent storage at − 80 °C as soon as required for analysis.

### Cell culture and reagents

The normal oral keratinocytes HOK cells obtained from Tongpai (Shanghai, China) and human HNSCC cell lines (CAL-27, FaDu, and HN4) acquired from the American Type Culture Collection (ATCC) were cultured in Dulbecco's modified Eagle's medium (DMEM; GIBCO, USA), supplemented with 10% fetal bovine serum (FBS; GIBCO, USA) and 1% penicillin–streptomycin (Beyotime, China), under a humidified atmosphere of 5% CO_2_ at 37 °C. LY294002 (PI3K/AKT inhibitor) was ordered from MCE (Shanghai, China).

### Lentiviral vectors and cell transfection

The target sequences were synthesized by Genechem (Shanghai, China), including two lentivirus-based short hairpin RNAs (shRNAs; shP4HA2#1 and shP4HA2#2), a lentiviral overexpression vector for P4HA2 and corresponding controls. The shRNA sequences targeting P4HA2 (shRNA#1 sequence: 5ʹ-GCACATGACTGACCTGATTTA-3ʹ; shRNA#2 sequence: 5ʹ-GGGAACTTCCAGGAACCAAGT-3ʹ). In particular, cell culture was maintained in 6-well plates (1 × 10^5^ cells/well) to a confluence of 20–30%. HiTransG P infection-enhancing solution was used for transfecting Lentivirus into the cells at a 10 multiplicity of infection (MOI). Just after a 24-h period, the cells went through incubation in DMEM with 10% FBS and 2 μg/mL puromycin supplements for 1 week, after which a selection process was carried out. The extraction of RNA and protein was conducted to evaluate the transfection efficiencies.

### Real-time quantitative PCR (RT-qPCR)

TRIzol reagent (Takara, Japan) was utilized for extracting the total RNA from the cells, which were then reversely transcripted to cDNA through PrimeScript™ RT reagent Kit (Takara, Japan). The RT-qPCR was applied through SYBR Green PCR Master Mix (Takara, Japan) following the protocols. Using the 2^−ΔΔCt^ method, the relative gene expressions were calculated, with normalization to GAPDH expression. Table 1 lists the RT-qPCR primer sequences.

**Table 1:** The primers utilized for RT-qPCR analysis of mRNA levels

Target ID	Primer sequence, 5ʹ –3ʹ
P4HA2	F: CAAACTGGTGAAGCGGCTAAA
	R: GCACAGAGAGGTTGGCGATA
GAPDH	F: CACCCACTCCTCCACCTTTG
	R: CCACCACCCTGTTGCTGTAG

### Western blot analysis

The total cellular protein amount was evaluated via the BCA protein quantification kit (Beyotime, China) subsequent to their extraction with RIPA lysis buffer (Beyotime, China). Subsequently, the protein samples went through separation utilizing a 10% SDS–polyacrylamide gel (Beyotime, China). Following denaturation, the samples were subsequently transferred onto PVDF membranes (Bio-Rad, USA) which were then blocked with 5% skim milk and subjected to 4 °C overnight treatment with primary antibodies as follows: P4HA2 (1:1000, ab233197, Abcam); E-cadherin (1:1000, ab40772, Abcam); Vimentin (1:1000, ab92547, Abcam); N-cadherin (1:1000, ab18203, Abcam); PI3K (1:1000, ab302958, Abcam); p-PI3K (1:300, ab182651, Abcam); AKT (1:500, ab8933, Abcam); p-AKT (1:500, ab38449, Abcam); GADPH (1:1000, ab8245, Abcam). Following a 1-h incubation in the presence of secondary antibodies, all bands were measured utilizing an enhanced chemiluminescence (ECL) system kit (Bio-Rad, USA). The protein levels of GAPDH were utilized as loading controls, and densitometric analyses were conducted using ImageJ software. Relative quantification was performed after normalizing to the band intensities of GAPDH. A Mann–Whitney test was employed to evaluate the differences in protein expression between groups.

### Cell proliferation assays

The Cell Counting Kit-8 (CCK-8) was utilized for determining cell proliferation viability. Cells were digested before being planted in 96-well plates (1000 cells/well). Cells were grown for 0, 24, 48, 72, and 96 h before being treated with CCK-8 (10 µl /well) and incubated for 1 h at 37 °C. Subsequently, the absorbance measurement was conducted at 450 nm utilizing a microplate reader.

A colony formation assay was utilized to evaluate the cell viability. The HNSCC cells were digested with 0.25% trypsin solution and seeded into a 6-well plate at a density of 500 cells/well. Following a two-week culturing period, the cells were fixed using a 4% paraformaldehyde solution. A 0.5% crystal violet solution was prepared by dissolving crystal violet in methanol, which was then diluted in PBS to a 0.1% solution. The discernible cell colonies within each well stained with a 0.1% crystal violet solution for 15 min to facilitate visualization and enumeration of the colonies.

### Cell apoptosis assay

The process of flow cytometry was executed to examine apoptosis in accordance with standard protocols. Following two washes with PBS, cellular apoptosis was assessed through flow cytometry (BECKMAN COULTER, USA) utilizing the Annexin V-FITC/PI apoptosis detection kit (Beyotime, China).

### Transwell assay

Transwell assays were categorized into two distinct types, namely transwell migration and transwell invasion. For the experiment involving cell invasion was conducted utilizing 24-well transwell invasion chambers, which were equipped with Matrigel-coated membranes (Corning, USA). In brief, 3 × 10^4^ cells in a serum-free medium were introduced into the upper chambers while introducing 10% FBS media into the lower chamber. Following a 24-h incubation period, the cells penetrated the membrane filter were subjected to fixation, staining with 0.5% crystal violet, and subsequently counting through microscopic observation. To measure cell migration, a transwell chamber lacking Matrigel coating was employed. The cell migration assay was conducted similarly to that of the invasion assay.

### Wound healing assay

Seeding of the CAL-27 cells was performed in 6-well plates with incubating for 24 h at 37 °C. The wounds were made by scraping the cell layers with 100 µL plastic pipette tips. The distances of cell migration were quantified and captured through microscopic imaging (Olympus, Japan) at two-time points: 0 and 24 h post-scratch generation.

### Statistical analysis

The statistical analyses were performed with SPSS 22.0 software, reporting the data in the form of mean ± standard deviation (SD). Each experiment was repeated at least 3 times with comparable results. R version 3.6.3 was used for performing the bioinformatics analysis. The statistical analysis utilized in this study involved the implementation of the Student's *t* test for comparing two groups and the utilization of analysis of variance (ANOVA) for comparing multiple groups. *p* < 0.05 indicated a significant difference.

## Results

### P4HA2 is overexpressed in HNSCC and may act as a precise diagnosis and prognosis marker

**Fig. 1:**
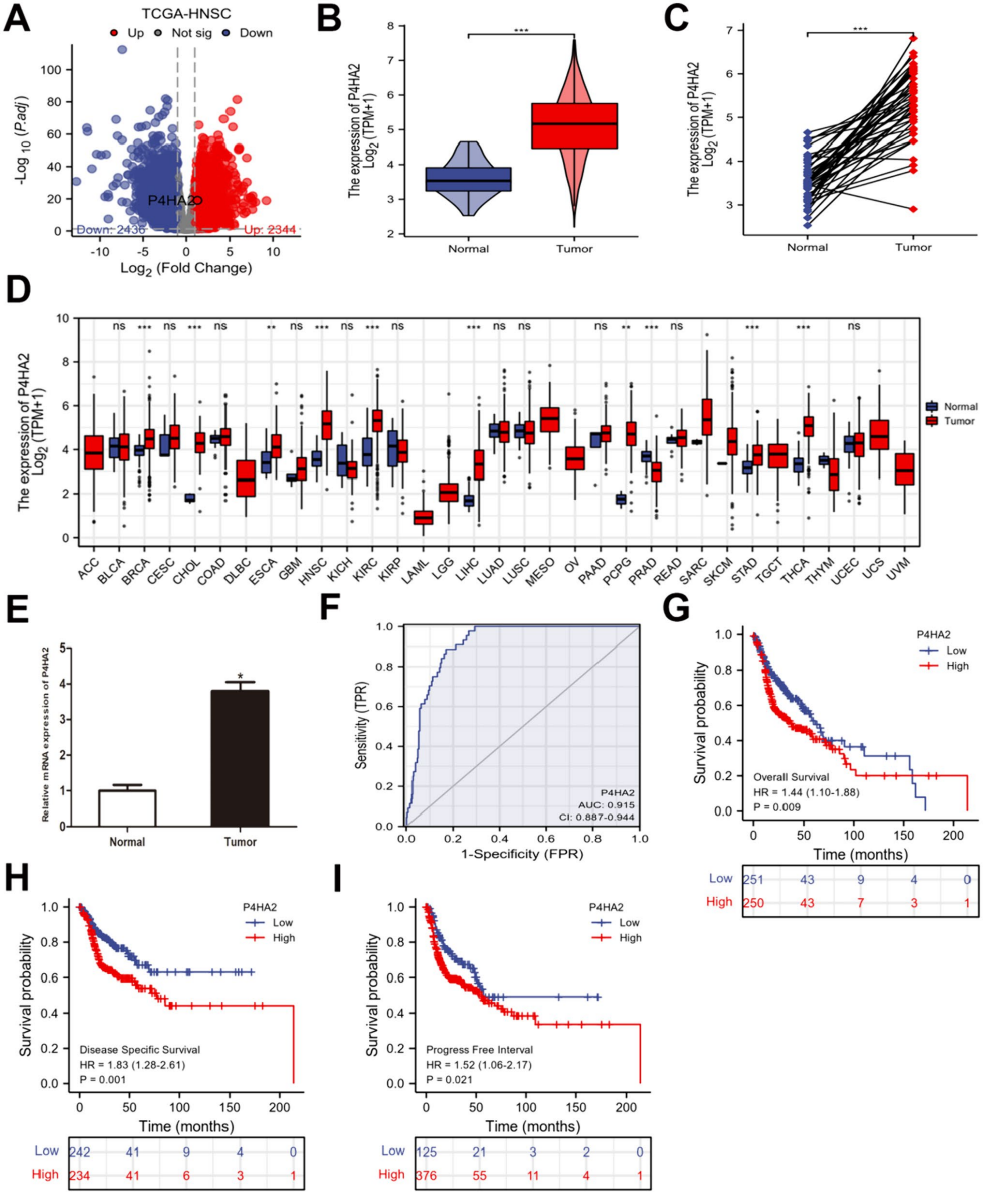
P4HA2 expression and its prognostic and diagnostic value in HNSCC patients in TCGA. **A** Volcano plot of the differentially expressed genes (DEGs). **B**, **C** In 504 HNSCC tissues alongside 44 non-malignant tissues and in 44 HNSCC tissues alongside their corresponding neighboring non-cancerous tissues, P4HA2 was overexpressed in HNSCC. **D** The different P4HA2 expression in multiple cancers. **E** The RT–qPCR of HNSCC tissues alongside adjacent non-cancerous tissues. **F** The ROC curve of diagnosis utilized for distinguishing HNSCC tissues from normal tissues. **G**–**I** The overall survival (OS), disease-specific survival (DSS), and progression-free interval (PFI) survival curves for distinguishing HNSCC tissues from normal tissues. *p < 0.05; **p < 0.01; ***p < 0.001; *ns* no statistical difference

When applying the criterion of an adjusted *p* value of 0.05 and |log2FC|> 1, 4780 differentially expressed genes (DEGs) from 19,578 genes were screened (2344 overexpressed and 2436 downregulated) (Fig. 1A). Among these genes, we have identified 83 up-regulated genes that demonstrate a significant association with reduced overall survival (OS), disease-specific survival (DSS), and progression-free interval (PFI) based on the criterion of HR > 1. The P4HA2 gene was obtained from the intersecting genes and subsequently subjected to further analysis (Figure S1). Based on TCGA data, P4HA2 was significantly overexpressed in HNSCC tissue, in contrast to neighboring non-malignant tissue (Fig. 1B, C). Furthermore, multiple malignancies had a considerable increase in P4HA2 contents compared to matched control tissues (Fig. 1D). Following that, the RT-qPCR results of HNSCC specimens revealed that P4HA2 levels in tumors were higher compared to non-cancerous tissues (Fig. 1E). The predictive P4HA2 efficiency on HNSCC was evaluated using a ROC analysis, with an estimated AUC of 0.915 (Fig. 1F). The Kaplan–Meier survival curves revealed that HNSCC patients having overexpressed P4HA2 exhibited a worse OS (Fig. 1G), a lower DSS (Fig. 1H), and a shorter PFI (Fig. 1I).

### P4HA2 suppresses apoptosis and enhances the growth of HNSCC in vitro

**Fig. 2:**
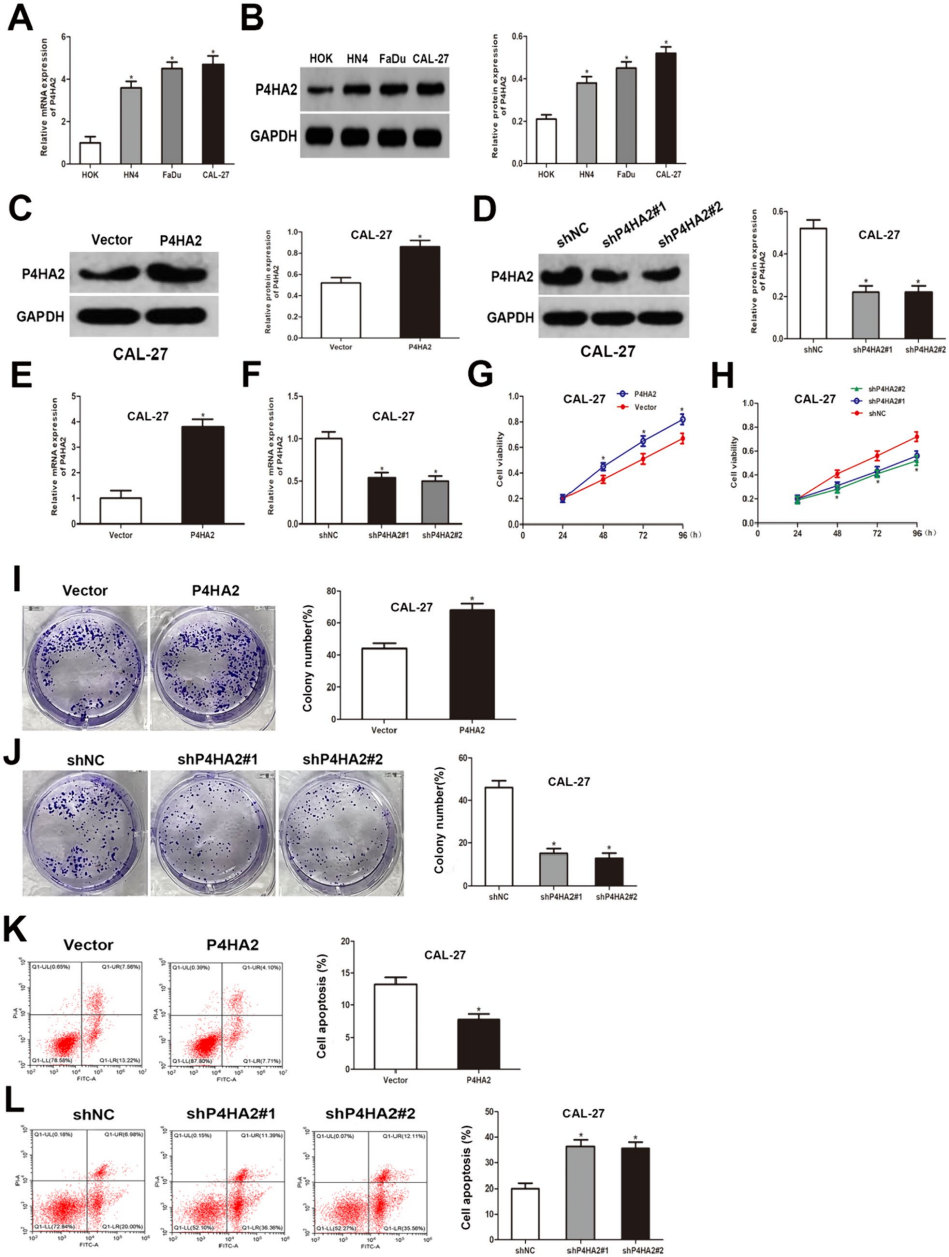
P4HA2 suppresses apoptosis and enhances HNSCC growth in vitro. **A**, **B** The RT–qPCR and western blotting of P4HA2 levels in the CAL-27, FaDu, HN4, and HOK cell lines. **C**, **D** The western blotting of P4HA2 levels in the CAL-27 cells upon transfection. **E**, **F** The RT-qPCR of P4HA2 levels in the CAL-27 cells upon transfection. **G**, **H** Cell viability based on the CCK-8 assay. **I**, **J** Colony formation assay outcomes for the multiplicative potential of CAL-27 cells. **K**, **L** The rate of cell apoptosis based on flow cytometry. *p < 0.05

P4HA2 levels were measured in three HNSCC cell lines (HN4, FaDu, and CAL-27) and normal human oral keratinocyte cells (HOK). P4HA2 levels were shown to be raised in all HNSCC cells, notably in CAL-27 cells (Fig. 2A, B). To assess the functional significance of P4HA2 in promoting HNSCC cell progression in vitro, lentiviral infection was employed to establish stable overexpression and knockdown of P4HA2 in CAL-27 and FaDu cells, respectively. The efficacy of both overexpression and knockdown was confirmed at the protein expression level as well as mRNA levels (Fig. 2C–F; Figure S2A-D). The impact of P4HA2 on cellular proliferation in HNSCC cells was evaluated through the utilization of CCK-8 and colony formation assays. As a result, the cell viability of HNSCC cells that went through transfection with sh-P4HA2 was comparatively lower than that of the shNC group; however, the P4HA2 overexpression group exhibited higher cell viability compared to the vector group (Fig. 2G, H; Figure S2E, F). Similarly, colony formation in vitro was revealed to exhibit an enhancement in the overexpressed P4HA2 cells while an inhibition in shP4HA2 transfected cells (Fig. 2I, J). According to flow cytometry results, overexpression of P4HA2 significantly reduced the apoptotic nuclei number; this effect can be counteracted by the inhibition of P4HA2 expression (Fig. 2K, L). These results collectively suggest that P4HA2 can inhibit apoptosis and enhance HNSCC proliferation.

### P4HA2 promotes the HNSCC cell migration and invasion in vitro

**Fig. 3:**
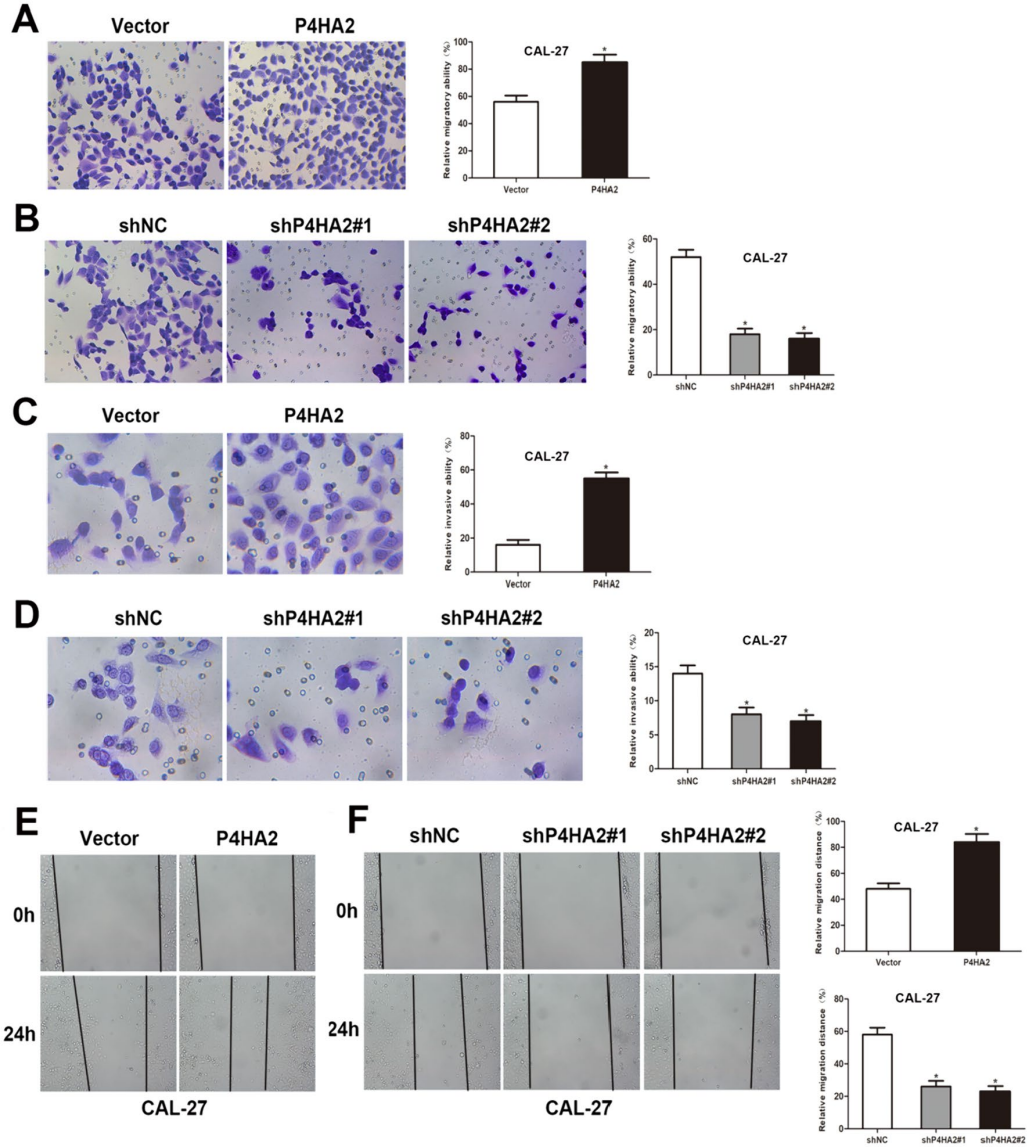
P4HA2 enhances HNSCC migration and invasion**. A**, **B** The CAL-27 cell migration capacity as tested by transwell migration assessment. **C**, **D** The CAL-27 cell invasion capacity as tested by transwell invasion assay. **E**, **F** The CAL-27 cell migratory capacity as tested by wound-healing assay. *p < 0.05

For assessing the P4HA2 influences on HNSCC migratory and infiltrative abilities, both wound healing and transwell assays were utilized. Based on the transwell migration assay, P4HA2 upregulation promoted HNSCC cell migration, while P4HA2 downregulation reduced it (Fig. 3A, B). In addition, transwell invasion experiments revealed that up-regulating P4HA2 encouraged cell invasion, while down-regulating P4HA2 inhibited it (Fig. 3C, D; Figure S2G, H). As seen in wound healing assays, P4HA2 over-expression enhanced HNSCC cell migration, while P4HA2 knockdown significantly decreased it (Fig. 3E, F). The above findings revealed that P4HA2 enhanced HNSCC cell invasion and migration.

### P4HA2 co-expressed genes and functional enrichment analysis in TCGA- HNSCC patients

**Fig. 4:**
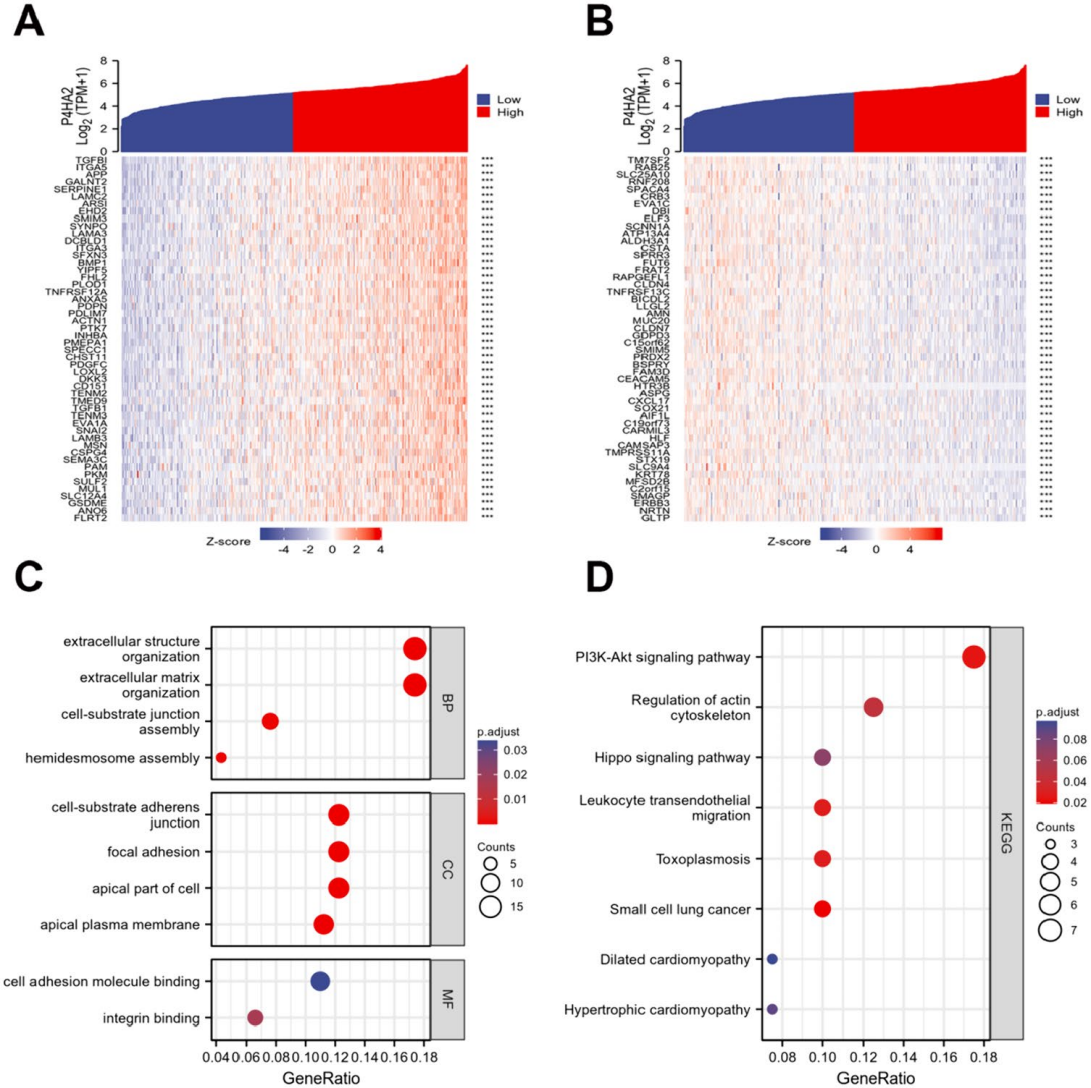
P4HA2 co-expressed genes and functional enrichment analysis in TCGA- HNSCC patients. **A** The top 50 genes exhibiting a positive correlation to P4HA2 in HNSCC are illustrated in the heatmap. **B** The top 50 genes exhibiting a negative correlation to P4HA2 in HNSCC are illustrated in the heatmap. **C**, **D** GO and KEGG pathway analyses of the top 50 genes exhibiting a positive and negative correlation to P4HA2 in HNSCC. ***p < 0.001

Next, co-expressed P4HA2 genes were identified in HNSCC through the TCGA data to understand the role of P4HA2 in HNSCC better. The heatmap displays the top 50 genes exhibiting a positive and negative correlation to P4HA2 in HNSCC (Fig. 4A, B), which were assessed by conducting the GO and KEGG pathway analyses to fully grasp the probable functions and molecular mechanisms. The GO analysis indicated a main enrichment in extracellular structure organization and extracellular matrix organization (Fig. 4C), and based on the KEGG analysis, the enrichment was mainly observed in the PI3K/AKT pathway (Fig. 4D).

### P4HA2 functions via modulating EMT and PI3K/AKT pathway in HNSCC

**Fig. 5:**
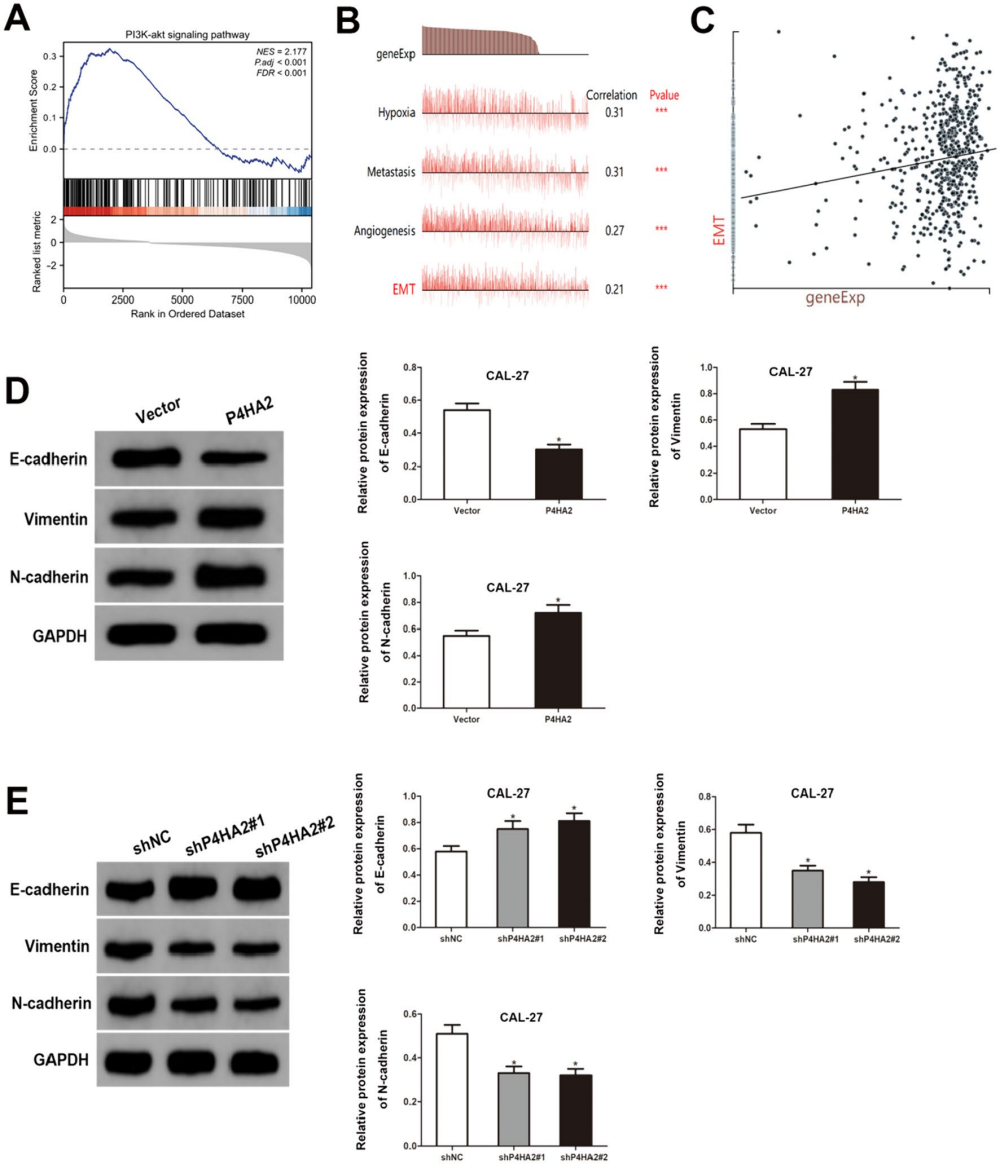
P4HA2 functions via modulating EMT and PI3K/AKT pathway in HNSCC cells. **A** GSEA provided a correlation between P4HA2 expression and PI3K/AKT pathway activation. **B**, **C** P4HA2 was positively connected with regulating EMT, according to data from CancerSEA. **D**, **E** E-cadherin, Vimentin, and N-cadherin protein levels in CAL-27 cells evaluated by western blotting. *p < 0.05; ***p < 0.001

According to the TCGA data, the GSEA analysis revealed a significant enrichment of the PI3K/AKT pathway in the HNSCC samples exhibiting high P4HA2 expression (Fig. 5A). To investigate the functional states of various malignancies based on P4HA2 expression levels, a single-cell analysis was conducted utilizing CancerSEA (Figure S3), revealing that P4HA2 expression level has a positive correlation to the EMT in HNSCC (Fig. 5B, C). In addition, the EMT marker levels were assessed via western blot analysis, indicating that P4HA2 overexpression decreased E-cadherin levels while increasing Vimentin and N-cadherin protein levels in HNSCC (Fig. 5D), which was contrary to the P4HA2 knockdown effect (Fig. 5E).

### P4HA2 promotes proliferation, migration, invasion, and EMT by modulating the PI3K/AKT pathway in HNSCC cells

**Fig. 6:**
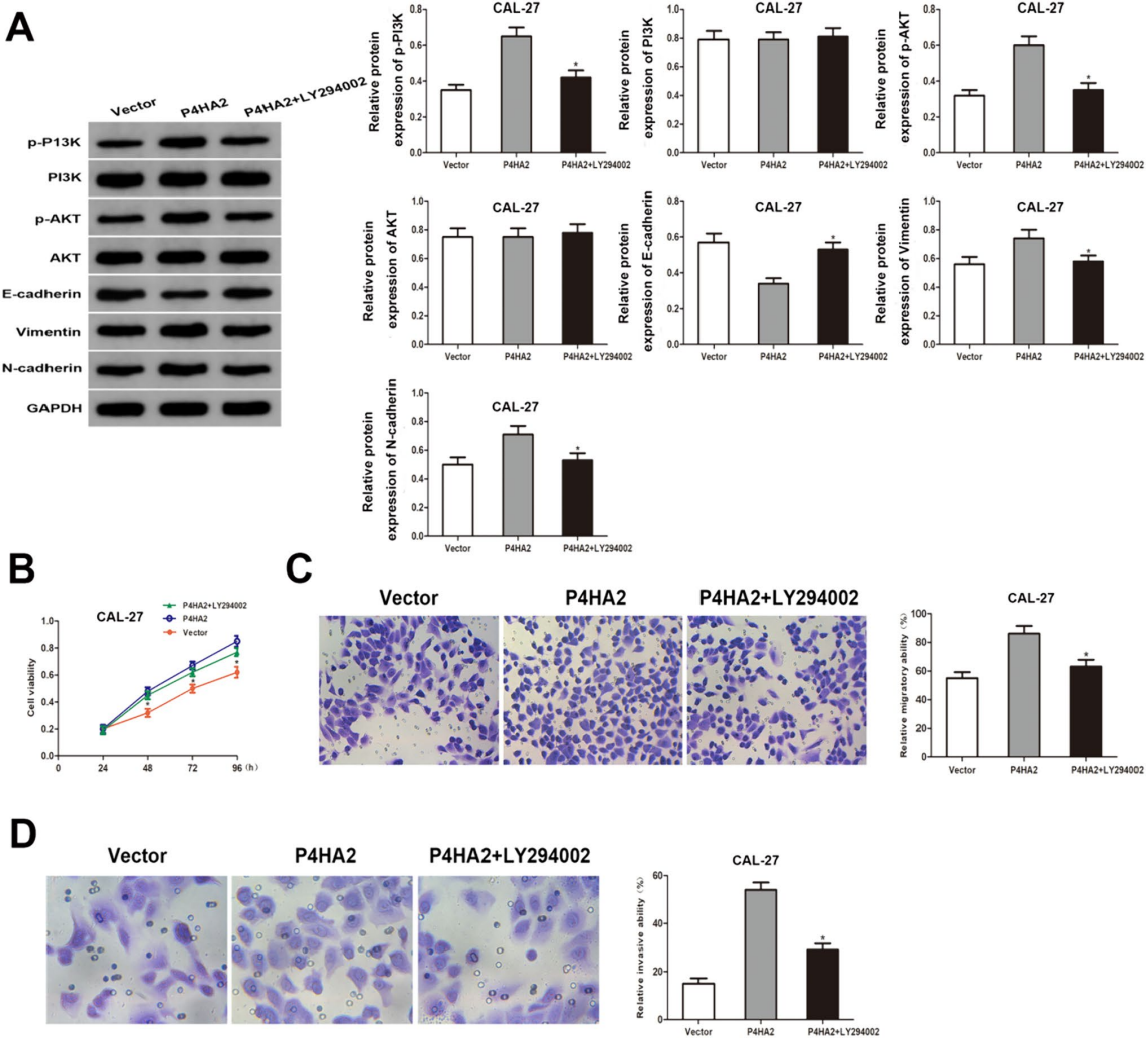
P4HA2 promotes proliferation, migration, invasion, and EMT by modulating the PI3K/AKT signaling pathway in HNSCC cells. The CAL-27 cells of the P4HA2 + LY294002 group followed the LY294002 (10 μM) treatment. **A** Protein expression levels of PI3K, p-PI3K, AKT, p-AKT, E-cadherin, Vimentin, and N-cadherin in CAL-27 cells. **B**–**D** LY294002 attenuated the P4HA2 overexpression-induced CAL-27 cell proliferation, migration, and invasion based on the CCK-8, transwell migration, and transwell invasion assays. *p < 0.05

Subsequently, LY294002, a PI3K/AKT pathway inhibitor, was utilized to provide additional evidence of the P4HA2 effect on activating the PI3K/AKT pathway in HNSCC. Following pre-treating CAL-27 cells with 10 μM LY294002 for 1 h, changes were observed in the associated molecules. Based on western blot analysis, P4HA2 overexpression in HNSCC cells raised p-PI3K, p-AKT, Vimentin, and N-cadherin levels while reducing E-cadherin levels. The PI3K/AKT inhibitor LY294002 prevented P4HA2-induced PI3K phosphorylation, AKT phosphorylation, and EMT (Fig. 6A). Additionally, LY294002 was found to significantly reverse P4HA2 overexpression effects on HNSCC proliferation, migration, and invasion (Fig. 6B–D).

## Discussion

Although HNSCC is very curable if detected at an early stage, most individuals are identified at a somewhat late stage, which leads to a poor prognosis for HNSCC patients [14]. Due to their poor treatment response and significant drug resistance, HNSCC negatively affects the patient's quality of life to a great extent [15]. The efficiency of diagnostic and therapeutic approaches in the early HNSCC stages can be increased by the discovery of novel molecular biomarkers [16]. Therefore, there is a crucial need to explore efficient diagnostic and therapeutic biomarkers for HNSCC patients.

P4HA2 is frequently observed to exhibit expression in fibroblasts and assists in collagen maturation through hydroxylating procollagen proline residues. Collagen, the primary component of the ECM, is important in tumor growth [17]. P4HA2 was shown to have a correlation to poor prognosis in various malignancies. It has been demonstrated that P4HA2, which has an association with a poor prognosis, enhances breast cancer growth and metastasis by controlling collagen deposition [18]. According to Cao et al. [10], P4HA2 functions as an oncogenic agent by inducing EMT in cervical cancer cells, thereby enhancing their motility, invasion, and proliferation. Lin et al. [19] reported that P4HA2 functions as an oncogenic molecule in glioma malignancies through modulating collagen expressions and the downstream PI3K/AKT pathway. However, P4HA2 biological role in HNSCC and the mechanisms behind it are still poorly understood. In the present study, the bioinformatic analysis revealed elevated P4HA2 levels in human HNSCC tissues contrary to non-cancerous tissues. In addition, a decreased survival time and a poorer prognosis were associated with elevated P4HA2 expression in HNSCC, suggesting that P4HA2 may aid in the growth of HNSCC. The findings of the experimental validation matched those of the bioinformatic study. The data revealed that the overexpressed P4HA2 increased HNSCC proliferation, migration, and invasion while suppressing apoptosis, whereas P4HA2 silencing resulted in contrasting effects.

Cancer metastasis, the primary cause of mortality associated with cancer, is a biological process whereby cells leave the initial tumor and grow into a new tumor in a different place [20]. EMT, a process by which epithelial cells take on a mesenchymal character, is crucial for cancer migration [21]. As a result, an investigation was conducted to analyze the expression of three genes associated with EMT (E-cadherin, Vimentin, and N-cadherin) to understand the P4HA2 role in the EMT of HNSCC better. It was found through bioinformatics analysis and experimental validation that P4HA2 overexpression increased EMT while P4HA2 knockdown inhibited EMT.

Prior research has suggested how PI3K-AKT is crucial in HNSCC, as it was found that the PI3K-AKT is hyperactive in 90% of HNSCC cases and was observed to govern various aspects of tumor biology, such as proliferation, metabolism, inflammation, motility, and metastasis [22–24]. The findings of our study indicate that P4HA2 overexpression in HNSCC increased PI3K/AKT phosphorylation, which suggests that the PI3K/AKT axis contributes to the cancer-promoting impacts of P4HA2 overexpression in HNSCC. In addition, it was observed that the LY294002 inhibitor of the PI3K/AKT pathway restored the effect of the overexpressed P4HA2 on HNSCC proliferation, migration, invasion, and EMT.

## Conclusion

To conclude, we revealed that P4HA2 was overexpressed in HNSCC and had the potential to enhance HNSCC proliferation, migration, invasion, and EMT by activating the PI3K/AKT pathway. Thus, P4HA2 can be a useful biomarker in identifying and predicting the prognosis of HNSCC patients.

### Supplementary Information

Below is the link to the electronic supplementary material.

Supplementary file1 (DOCX 2280 kb)

## Data Availability

The data used and analyzed during the current study are available from the corresponding author on reasonable request.
