# Supplementary material for: P4HA2 contributes to head and neck squamous cell carcinoma progression and EMT through PI3K/AKT signaling pathway
Source: Med Oncol. 2024 May 23;41(6):163. doi: 10.1007/s12032-024-02358-w (PMC11111551; doi:10.1007/s12032-024-02358-w)
Supplement: Supplementary file 1 — Supplementary file1 (DOCX 2280 kb) [file 12032_2024_2358_MOESM1_ESM.docx]

**
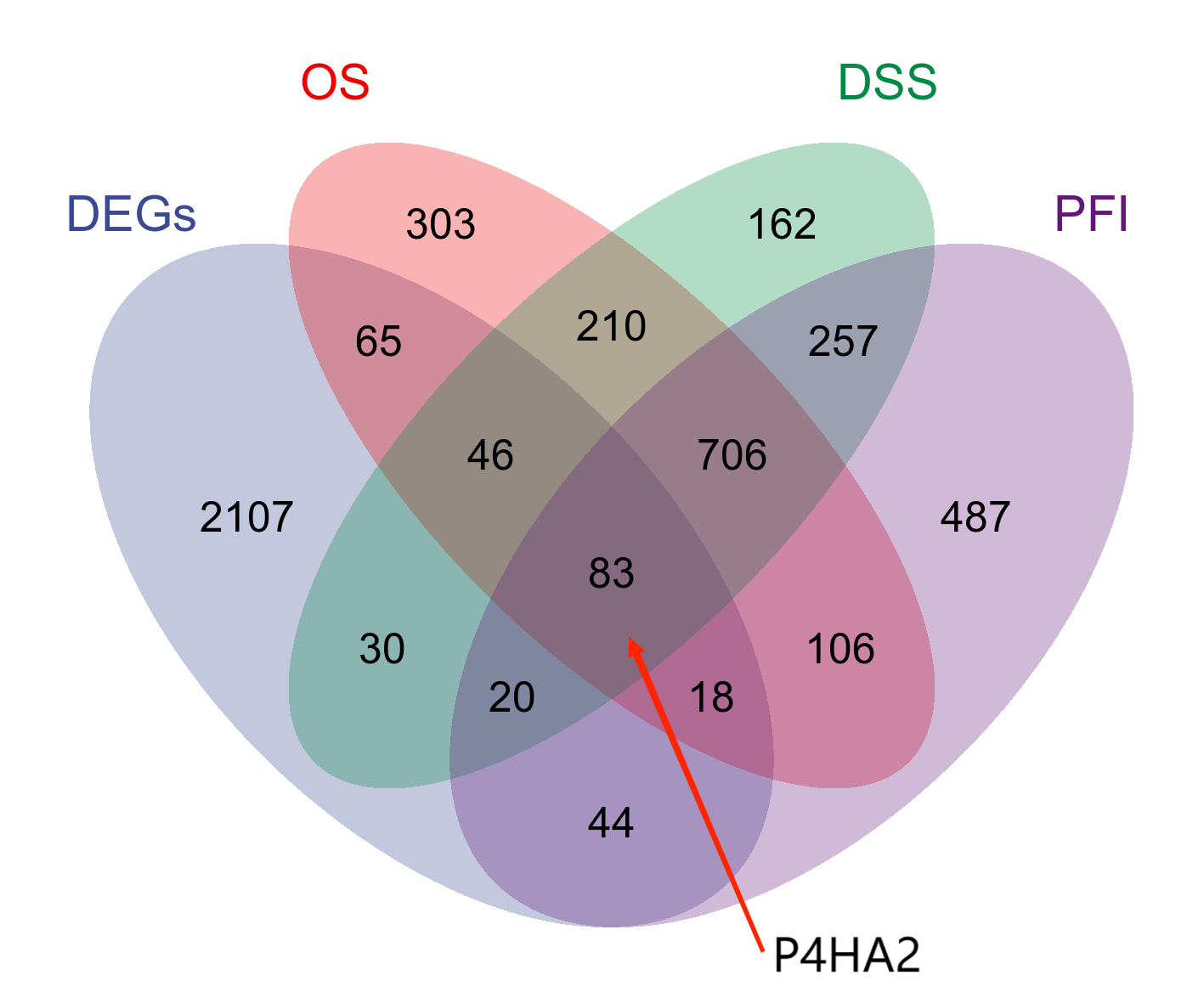
**

**Figure S1. The Venn diagram was employed to identify the overlapping genes among DEGs, genes associated with poor OS, genes associated with poor DSS, and genes associated with poor PFI.**

**
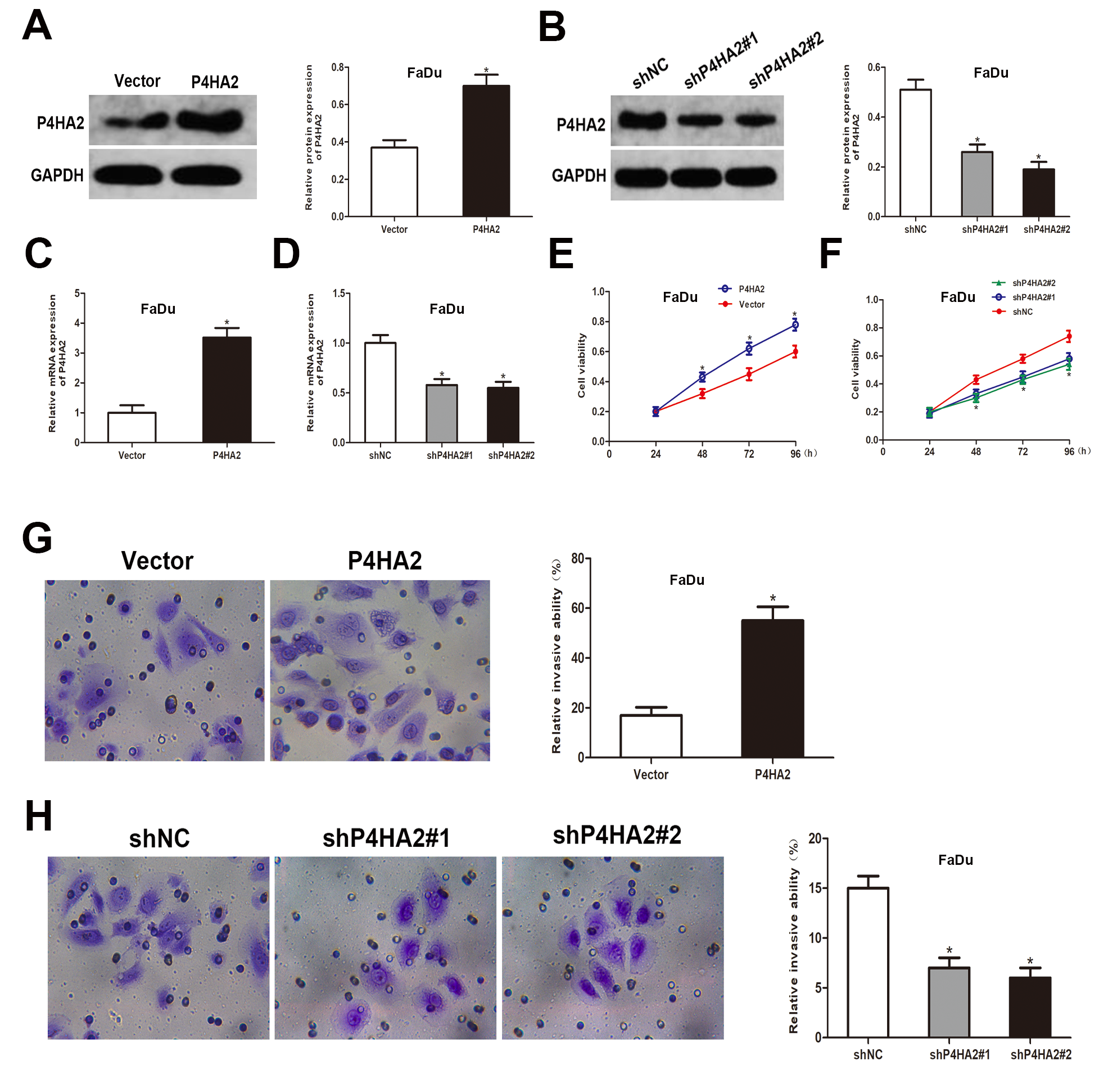
**

**Figure S2.** **P4HA2 enhances HNSCC growth and invasion in vitro.** (A, B) The western blotting of P4HA2 levels in the FaDu cells upon transfection. (C, D) RT-qPCR assessment of P4HA2 levels in the FaDu cells upon transfection. (E, F) Cell viability based on the CCK-8 assay. (G, H) The FaDu cell invasion capacity as tested by transwell invasion assay.

**
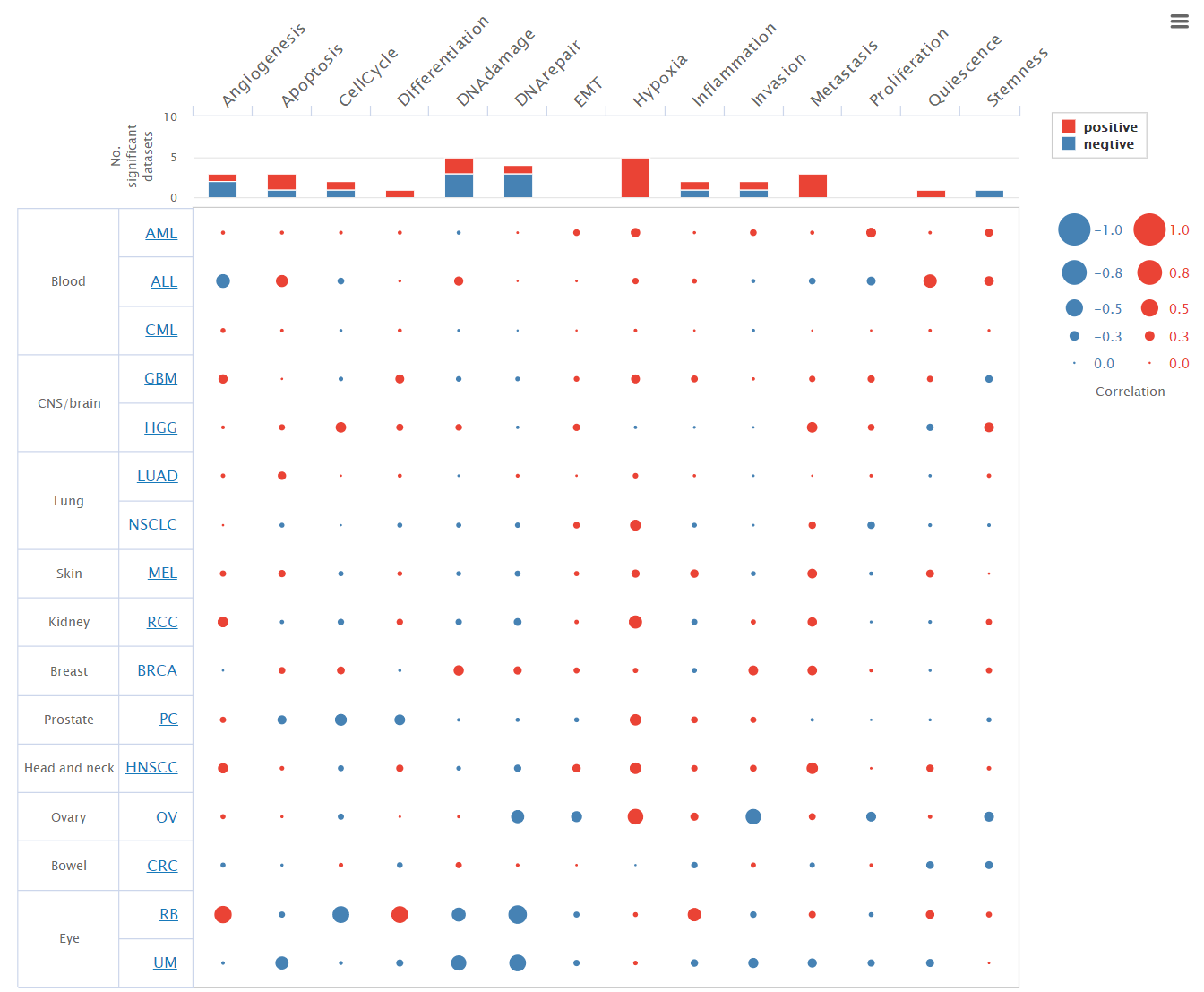
**

**Figure S3. Functional status of P4HA2 as determined from single cell analysis according to the CancerSEA database for 16 different cancer types.** P4HA2 was favorably connected to the functional states, as shown by the red plots, whereas P4HA2 has a negative correlation to the functional states, as shown by the blue plots.
